# Preoperative N-terminal pro-B-type natriuretic peptide and myocardial injury after stopping or continuing renin–angiotensin system inhibitors in noncardiac surgery: a prespecified analysis of a phase 2 randomised controlled multicentre trial

**DOI:** 10.1016/j.bja.2024.01.010

**Published:** 2024-02-09

**Authors:** Ana Gutierrez del Arroyo, Akshaykumar Patel, Tom E.F. Abbott, Salma Begum, Priyanthi Dias, Sameer Somanath, Alexander Middleditch, Stuart Cleland, David Brealey, Rupert M. Pearse, Gareth L. Ackland, the SPACE trial investigators, Gareth Ackland, Tim Martin, Maria Fernandez, Fatima Seidu, Mari-Liis Pakats, Otto Mahr, Neil MacDonald, Filipa Dos Santos, Amaia Arrieta Garcia, Ruzena Uddin, Salma Begum, Rupert Pearse, Emily Subhedar, Yize Wan, Akshaykumar Patel, Tasnin Shahid, Mevan Gooneratne, Charlotte Trainer, Bethan Griffiths, Steven Dunkley, Shaun May, Sophie Walker, Alexander Fowler, Timothy Stephens, Monica Oliveira, Marta Januszewska, Edyta Niebrzegowska, Vanessa Amaral, Jamila Kassam, Sophie Young, Shanaz Ahmad, Jan Whalley, Ryan Haines, Sara Hui, Rob Hammond, David Crane, David Brealey, Sohail Bampoe, Robert Stephens, Anna Reyes, Gladys Martir, Chimverly Diaz, Stuart Cleland, Gary Minto, Natasha Wilmshurst, Debbie-Claire Affleck, Tracy Ward, Gavin Werrett, Susan Cummins, Alan Amber, Andrew Biffen, Stephen Boumphrey, Elizabeth Cann, Charlotte Eglinton, Elaine Jones, Memory Mwadeyi, Sam Piesley, Richard Cowan, Julie Alderton, Fiona Reed, Joanne Smith, Amy Turner, Lorraine Madziva, Abigail Patrick, Penny Harris, Harry Lang, Alexander Middleditch, Anthony Pickering, Catherine O’Donovan, Rebecca Houlihan, Rosina Jarvis, Andrew Shrimpton, Toni Farmery, Katy Tucker, Danielle Davis, Sameer Somanth, Louise Duncan, Helen Melsom, Sarah Clark, Melanie Kent, Michelle Wood, Ami Laidlaw, Tracy Matheson-Smith, Kathryn Potts, Andrea Kay, Stefanie Hobson, John Sear, Vikas Kapil, Andrew Archbold, Matt Wilson, Drilona Dndrejaj, Dennis Ly, Akshaykumar Patel

**Affiliations:** 1 Translational Medicine and Therapeutics, William Harvey Research Institute, Queen Mary University of London, London, UK; 2 County Durham and Darlington NHS Foundation Trust, Durham, UK; 3 University Hospitals Bristol NHS Foundation Trust, Bristol, UK; 4 University Hospitals Plymouth NHS Trust, Plymouth, UK; 5 Bloomsbury Institute of Intensive Care Medicine, University College London, London, UK; 6 UCL Hospitals NHS Foundation Trust, London, UK

**Keywords:** major cardiac events, myocardial injury, noncardiac surgery, perioperative care, renin-angiotensin system inhibitors

## Abstract

**Background:**

Patients with elevated preoperative plasma N-terminal pro-B-type natriuretic peptide (NT-proBNP >100 pg ml^−1^) experience more complications after noncardiac surgery. Individuals prescribed renin–angiotensin system (RAS) inhibitors for cardiometabolic disease are at particular risk of perioperative myocardial injury and complications. We hypothesised that stopping RAS inhibitors before surgery increases the risk of perioperative myocardial injury, depending on preoperative risk stratified by plasma NT-proBNP concentrations.

**Methods:**

In a preplanned analysis of a phase 2a trial in six UK centres, patients ≥60 yr old undergoing elective noncardiac surgery were randomly assigned either to stop or continue RAS inhibitors before surgery. The pharmacokinetic profile of individual RAS inhibitors determined for how long they were stopped before surgery. The primary outcome, masked to investigators, clinicians, and patients, was myocardial injury (plasma high-sensitivity troponin-T ≥15 ng L^−1^ or a ≥5 ng L^−1^ increase, when preoperative high-sensitivity troponin-T ≥15 ng L^−1^) within 48 h after surgery. The co-exposures of interest were preoperative plasma NT-proBNP (< or >100 pg ml ^−1^) and stopping or continuing RAS inhibitors.

**Results:**

Of 241 participants, 101 (41.9%; mean age 71 [7] yr; 48% females) had preoperative NT-proBNP >100 pg ml ^−1^ (median 339 [160–833] pg ml^−1^), of whom 9/101 (8.9%) had a formal diagnosis of cardiac failure. Myocardial injury occurred in 63/101 (62.4%) subjects with NT-proBNP >100 pg ml^−1^, compared with 45/140 (32.1%) subjects with NT-proBNP <100 pg ml ^−1^ {odds ratio (OR) 3.50 (95% confidence interval [CI] 2.05–5.99); *P*<0.0001}. For subjects with preoperative NT-proBNP <100 pg ml^−1^, 30/75 (40%) who stopped RAS inhibitors had myocardial injury, compared with 15/65 (23.1%) who continued RAS inhibitors (OR for stopping 2.22 [95% CI 1.06–4.65]; *P*=0.03). For preoperative NT-proBNP >100 pg ml^−1^, myocardial injury rates were similar regardless of stopping (62.2%) or continuing (62.5%) RAS inhibitors (OR for stopping 0.98 [95% CI 0.44–2.22]).

**Conclusions:**

Stopping renin-angiotensin system inhibitors in lower-risk patients (preoperative NT-proBNP <100 pg ml ^−1^) increased the likelihood of myocardial injury before noncardiac surgery.

**Editor's key points**

• Elevated preoperative plasma NT-proBNP is associated with increased complications after noncardiac surgery.
• The impact of stopping renin-angiotensin system (RAS) inhibitors before surgery on the risk of perioperative myocardial injury in patients risk stratified by preoperative plasma NT-proBNP concentrations was determined in a substudy of the SPACE trial.
• For subjects with lower preoperative risk who stopped RAS inhibitors, risk of myocardial injury determined by plasma high-sensitivity troponin-T was 2.2-fold greater compared with subjects who continued RAS inhibitors.
• In subjects at high preoperative risk, myocardial injury rates were similar regardless of stopping or continuing RAS inhibitors.
• Preoperative discontinuation of renin–angiotensin system inhibitors in lower-risk patients undergoing noncardiac surgery increased the risk of myocardial injury or other perioperative complications.

Myocardial injury after noncardiac surgery (MINS)^1^^,^^2^ occurs frequently in patients with pre-existing cardiometabolic pathology.^3^ Myocardial injury, independent of symptoms or ECG changes,^2^ increases the risk of death and further cardiovascular complications, even after discharge from hospital.^4^ Surgical patients at most risk of postoperative complications^5^^,^^6^ are commonly prescribed angiotensin converting enzyme inhibitors (ACEi), angiotensin-II receptor blockers (ARB), or both to treat hypertension, chronic kidney disease, and cardiac failure.^7^, ^8^, ^9^ Renin–angiotensin system (RAS) inhibitors reduce organ injury, in part, by reducing systemic inflammation.^10^

Preoperative plasma N-terminal pro-B-type natriuretic peptide (NT-proBNP) is associated with myocardial injury and complications within 30 days after noncardiac surgery, over and above conventional clinical risk factors.^11^, ^12^, ^13^ The VISION investigators showed that preoperative NT-proBNP values >100 pg ml^−1^ were associated with at least a two-fold higher risk of sustaining myocardial injury, death, or both within 30 days of surgery in >10,000 patients aged 45 yr or older having inpatient noncardiac surgery.^11^ NT-proBNP is also used to guide therapy and estimate morbidity and mortality in heart failure.^14^ Withdrawal of ACEi and ARB during hospitalisation for heart failure is associated with more frequent readmission and death after discharge, even allowing for the severity of heart failure.^15^ Although RAS inhibitors are frequently stopped before surgery in the widely held belief that this prevents intraoperative hypotension and organ injury,^16^^,^^17^ the potential for adverse effects in patients with biochemical evidence of untreated subclinical cardiovascular dysfunction as indicated by high NT-proBNP concentrations is unclear. Current European^12^^,^^18^ and North American^19^^,^^20^ guidelines do not provide specific advice on this issue, despite recent studies such as METS demonstrating a substantial number of patients having high preoperative NT-proBNP concentrations consistent with occult heart failure.^13^^,^^21^

In a multicentre, randomised, open-label phase 2a trial in the UK, we found that stopping RAS inhibitors according to their individual pharmacokinetic profile failed to reduce myocardial injury but increased haemodynamic instability within 48 h of surgery.^22^ In this *a priori* analysis, we assessed whether patients randomised to stop or continue RAS inhibitors sustained different rates of myocardial injury and complications if they had preoperative NT-proBNP concentrations predictive of excess morbidity.

## Methods

### Study design

This study was a preplanned analysis of the Stopping Perioperative ACE-inhibitors/ARBs (SPACE) phase 2a Clinical Trial of an Investigational Medicinal Product (ISRCTN17251494), regulated by the Health Research Authority (UK) and the Medicines and Healthcare products Regulatory Agency UK (Eudract: 2016-004141-90). Six UK centres recruited patients who provided written informed consent, in accord with the London (City and East) research ethics committee (16/LO/1495). An independent steering committee and data monitoring and ethics committee (DMEC) oversaw the trial (Supplementary data).

### Inclusion criteria

• Adults prescribed RAS inhibitors aged ≥60 yr
• American Society of Anesthesiologists physical status ≥3
• Elective major surgery requiring general anaesthesia for procedures lasting ≥120 min

### Exclusion criteria

• Current participation in any other interventional clinical trial
• Myocardial infarction within the 3 months preceding surgery

### Randomisation

Patients were assigned randomly in a 1:1 ratio to either continue or discontinue RAS inhibitors according to the individual pharmacokinetics of each drug. Randomisation was performed centrally, with minimisation by centre, planned surgical procedure category, and RAS inhibitor type.

### Post-randomisation management of renin–angiotensin system inhibitors

RAS inhibitors were restarted after surgery on the morning of postoperative day 2 in accord with recommendations by European Society of Cardiology (ESC) guidelines^18^ at the usual dose. Restarting RAS inhibitors did not occur on postoperative day 2 if systolic arterial pressure was <90 mm Hg in the preceding 12 h or vasoactive therapy was used or if acute kidney injury occurred (KDIGO criteria).^23^ All other treatments were managed by the local clinical teams.

### Data collection

Blood samples were collected before induction of anaesthesia, 24 h, and 48 h after surgery (see Supplementary data for schedule of visits), from which serum and plasma was prepared. Plasma troponin samples were batch-analysed at the same laboratory (The Doctor's Laboratory, London, UK) by personnel masked to all study details. To minimise bias, follow-up data were collected by a study team member who was masked to the treatment group allocation.

### Explanatory measure: N-terminal pro-B-type natriuretic peptide

The VISION study found that in 10,402 subjects aged 45 yr or older having inpatient noncardiac surgery, preoperative NT-proBNP values >100 pg ml^−1^ were associated with adjusted hazard ratios for the composite outcome of vascular death and MINS within 30 days after surgery of at least 2.27 (95% confidence interval [CI] 1.90–2.70).^11^ Preoperative NT-proBNP values >100 pg ml^−1^ were also associated with a two- to four-fold increased risk of dying within 30 days of surgery.

### Measurement of N-terminal pro-B-type natriuretic peptide

Batched analysis of plasma samples stored at −80 °C was undertaken after the end of the study by personnel masked to all study details. We used a single-wash colorimetric sandwich enzyme-linked immunosorbent assay (ELISA) to quantify human NT-proBNP (ab263877; Abcam, Cambridge, UK) which has a sensitivity of 11.5 pg ml^−1^ and a 21.9–1400 pg ml^−1^ range. Plasma samples were measured in duplicate after 50% dilution to ensure that higher values associated with heart failure were quantified. Standard curves for each plate were constructed (mean *R*^2^=0.9963; Supplementary Fig. S1).

### Primary endpoint

The primary endpoint was the occurrence of myocardial injury, a binary variable derived from the same threshold values for high-sensitivity troponin-T (hsTnT) concentrations (5th generation assay, Roche, Rotkreuz, Switzerland) used by the VISION study (but without further adjudication of likely aetiology).^2^ A positive event was defined by plasma hsTnT ≥15 ng L^−1^ or a ≥5 ng L^−1^ increase, when preoperative hsTnT ≥15 ng L^−1^) within 48 h after surgery. The primary outcome required samples to be collected at each specified time point.

### Secondary endpoints

Consistent with the Standardized Endpoints Consensus for cardiovascular outcomes,^24^ we report myocardial injury and the rates of heart failure and myocardial infarction,^2^^,^^25^^,^^26^ the definitions for which are provided in Supplementary data.^24^ The secondary outcomes were: highest absolute concentration of troponin-T measured within 48 h of surgery; clinically-defined myocardial infarction within 30 days after randomisation; clinically-defined acute heart failure within 30 days after randomisation; clinically-defined stroke within 30 days after randomisation; infection within 30 days after randomisation (definitions provided in Supplementary data).

In accord with STEP-COMPAC guidelines, the core outcome set for trials in perioperative care is also presented,^27^ including mortality, length of hospital stay, unplanned readmission within 30 days, and EQ5D™, a standardised measure of health-related quality of life developed by the EuroQol Group to provide a simple, generic questionnaire for use in clinical and economic appraisal and population health surveys.

### Exposures of interest

We assessed whether stopping or continuing RAS inhibitors, in the presence or absence of low (<100 pg ml^−1^) or high (>100 pg ml ^−1^) preoperative plasma NT-proBNP, was associated with the incidence of myocardial injury.

### Statistical analysis

A statistical analysis plan was published online before the NT-proBNP analyses were undertaken (https://www.qmul.ac.uk/ccpmg/sops--saps/statistical-analysis-plans-saps/), with only the study statistician having access to the database. The original sample size for SPACE was 260 subjects (130 per trial group) based on the VISION-UK data showing that subjects >60 yr receiving RAS inhibitors had ∼40% incidence of elevated troponin as defined by VISION criteria. Accordingly, for power of 90%, a 20% absolute risk reduction in incidence of myocardial injury was sought (α=0.05), allowing for a 5% loss to follow-up rate. All analyses were performed with STATA (STATA 14, StataCorp 2015; Stata Statistical Software: Release 14; College Station, TX, USA), NCSS 2023 (NCSS 2023 Statistical Software (2023). NCSS, LLC; Kaysville, UT, USA), or both.

All analyses were by intention-to-treat. Summary statistics of the outcome by treatment group are presented with an estimated treatment effect and corresponding 95% CI (two-sided *P*-value). The primary and secondary outcomes were analysed by Fisher's exact test. For all other secondary outcomes with low event rates, no statistical analysis was performed. Absolute peak troponin concentrations were analysed using a mixed effects linear regression model, adjusted for the same covariates as the primary analysis and taking into account clustering by centre. In a *post hoc* analysis, after peer review, the relationships between continuous NT-proBNP, myocardial injury, and peak troponin were examined. We used NICE-based NT-proBNP thresholds that are used as indicators for, and guide the investigation of, heart failure (<400 ng L^−1^, 400–2000 ng L^−1^, >2000 ng L^−1^).^28^ The relationship with the primary outcome of stopping or continuing ACEi and ARBs in each NT-proBNP band was not formally tested.

## Results

**Fig 1:**
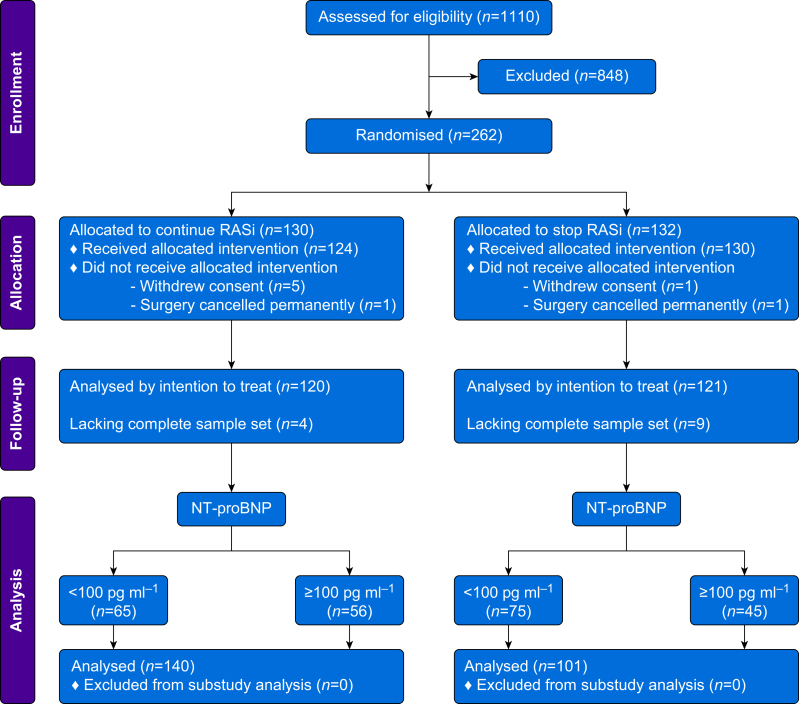
CONSORT diagram. Methods for screening and the determining of eligibility are provided in the Supplementary data. The primary outcome of significant high-sensitivity troponin-T (hsTnT) increase was not met if the preoperative sample was not collected or the day 1 or day 2 samples were also not collected. NT-proBNP, N-terminal pro-B-type natriuretic peptide; RASi, renin–angiotensin system inhibitor.

The first subject was randomised on July 31, 2017 and the trial completed recruitment on October 1, 2021 after 18 months of interrupted recruiting as a result of the COVID-19 pandemic. Of 1110 patients screened across six UK centres, 262 participants underwent randomisation before surgery, and 241/262 subjects had a complete set of serial blood samples after surgery (Fig. 1). The majority of subjects were hypertensive (97%) or were receiving treatment for diabetes mellitus (30.3%). Although a formal diagnosis of cardiac failure was recorded for 16/241 (6.7%), 108/241 (41.9%) participants (mean age 71 [7] yr; 48% females) had preoperative NT-proBNP ≥100 pg ml^−1^ (median 339 [25–75th centiles 160–833] pg ml^−1^; Table 1); 37/241 (15.4%) subjects had NT-proBNP values 400–2000 pg ml^−1^; and 7/241 (2.9%) subjects had NT-proBNP values >2000 pg ml^−1^ (Supplementary Fig. S2). The mean difference between preoperative NT-proBNP values for subjects stopping or continuing ACEi/ARBs was 254 pg ml^−1^ (95% CI −117 to 625 pg ml^−1^).

**Table 1:** Participant characteristics. All data mean (standard deviation) or *n* (%) (unless stated otherwise). COPD, chronic obstructive pulmonary disease; eGFR, estimated glomerular filtration rate. ∗eGFR derived from the CKD-EPI Creatinine Equation.^29^

	Preop NTproBNP <100 ng ml^−1^		Preop NTproBNP ≥100 ng ml^−1^	
	Continue	Stop	Continue	Stop
Number of subjects	65	75	56	45
Age (yr)	69 (6)	70 (6)	74 (7)	75 (7)
Female	32 (49)	38 (51)	24 (43)	20 (44)
Creatinine (mM)	85 (26)	84 (26)	90 (26)	107 (51)
Estimated GFR∗	56 (15)	53 (13)	56 (13)	47 (17)
Chronic kidney disease grade ≥3a	32 (49)	37 (49)	51 (91)	33 (73)
Haemoglobin (g L^−1^)	134 (15)	134 (12)	131 (17)	133 (18)
Chronic obstructive pulmonary disease	9 (14)	6 (8)	12 (21)	2 (4)
Asthma	11 (17)	7 (9)	4 (7)	6 (13)
Ischaemic cardiac disease	10 (15)	12 (16)	15 (27)	13 (29)
Diabetes mellitus	21 (32)	22 (29)	19 (34)	11 (24)
Cardiac failure	2 (3)	5 (7)	4 (7)	5 (11)
Cancer	15 (23)	20 (27)	16 (29)	10 (22)
Stroke	5 (7)	5 (7)	4 (7)	4 (9)
Peripheral vascular disease	1 (2)	4 (5)	6 (11)	4 (9)
Hypertension	63 (97)	73 (97)	54 (96)	44 (98)
Smoker	7 (11)	8 (11)	2 (4)	3 (7)
Angiotensin converting enzyme inhibitor	35 (54)	48 (64)	38 (68)	26 (58)
Angiotensin-II receptor blocker	30 (46)	27 (36)	18 (32)	19 (42)
Beta-blocker	13 (20)	18 (24)	23 (41)	19 (42)
Calcium channel blocker	21 (32)	29 (39)	21 (38)	14 (31)
Doxazosin	5 (8)	7 (9)	6 (11)	5 (11)
Diuretic	18 (28)	20 (27)	18 (32)	17 (38)
Statin	47 (72)	58 (77)	34 (61)	32 (71)
Nitrate	6 (9)	5 (7)	6 (11)	8 (18)
Antiplatelet agents	25 (38)	21 (28)	24 (43)	18 (40)
Anaesthetic technique				
General anaesthesia alone	19 (29)	21 (28)	16 (29)	11 (24)
General + epidural anaesthesia	9	9	7	9
General + spinal anaesthesia	14	20	16	9
General + other regional anaesthesia	10	15	7	5
Regional anaesthesia with sedation	5	5	5	7
Arterial pressure during surgery				
Systolic arterial pressure <90 mm Hg	32 (49)	29 (39)	22 (39)	11 (24)
Metaraminol during surgery (mg)				
Median (inter-quartile range)	3 (1–10)	3 (1–9)	3 (1–9)	5 (1–9)
Other vasopressor support	5 (8)	8 (11)	6 (11)	4 (9)

### Primary outcome: myocardial injury

**Fig 2:**
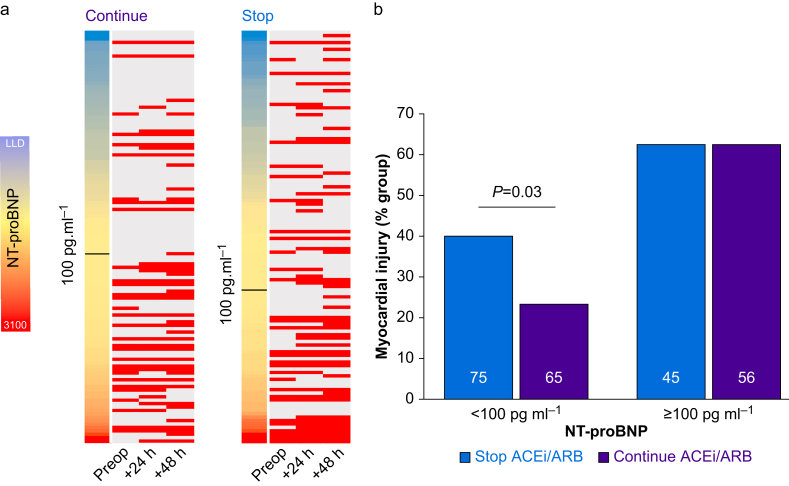
Primary outcome: myocardial injury. (a) Heat maps showing relationship between NT-proBNP concentration and presence or absence of myocardial injury in individual subjects randomised to stop or continue renin–angiotensin system (RAS) inhibitors. Multicoloured bar on left of each plot shows NT-proBNP scaled from lower limit of detection to maximal value (3100 pg ml^−1^). Each red bar represents high-sensitivity troponin-T (hsTnT) ≥15 ng L ^−1^. Black bars denote VISION-defined threshold for higher risk threshold of NT-proBNP ≥100 pg ml^−1^. (b) Myocardial injury occurred in 63/101 (62.4%) subjects with NT-proBNP ≥100 pg ml^−1^, compared with 45/140 (32.1%) subjects with NT-proBNP <100 pg ml^−1^ (odds ratio 3.50 [95% confidence interval 0.05–5.99]; *P*<0.0001). For subjects with preoperative NT-proBNP <100 pg ml ^−1^, 30/75 (40%) who stopped RAS inhibitors had myocardial injury, compared with 15/65 (23.1%) who continued RAS inhibitors (odds ratio for stopping 2.22; 95% confidence interval 1.06–4.65; *P*=0.03). For preoperative NT-proBNP ≥100 pg ml^−1^, myocardial injury rates were similar, regardless of stopping (62.2%) or continuing (62.5%) RAS inhibitors (odds ratio for stopping 0.98; 95% confidence interval 0.44–2.22). ACEi, angiotensin converting enzyme inhibitor; ARB, angiotensin-II receptor blocker; NT-proBNP, N-terminal pro-B-type natriuretic peptide.

Myocardial injury occurred in 63/101 (62.4%) subjects with NT-proBNP ≥100 pg ml^−1^, compared with 45/140 (32.1%) subjects with NT-proBNP <100 pg ml^−1^ (OR 3.50 [95% CI 2.05–5.99]; *P*<0.001). For subjects with preoperative NT-proBNP <100 pg ml^−1^, 30/75 (40%) who stopped RAS inhibitors had myocardial injury, compared with 15/65 (23.1%) who continued RAS inhibitors (OR [for stopping] 2.22; 95% CI 1.06–4.65; χ^2^=9.80; *P*=0.020, Fig. 2). For subjects with preoperative NT-proBNP ≥100 pg ml^−1^, myocardial injury rates were similar regardless of stopping (62.2%) or continuing (62.5%) RAS inhibitors (OR [for stopping] 0.98; 95% CI 0.44–2.22). The results were similar for 62 subjects with hsTnT ≥15 ng L^−1^ before surgery (31 in each group; Supplementary Fig. S3). The relationship between stopping or continuing RAS inhibitors across NT-proBNP bands (<400, 400–2000, >2000 pg ml^−1^) with the primary outcome was not formally tested (Supplementary Fig. S2).

### Secondary outcomes

#### Peak troponin T concentrations

**Fig 3:**
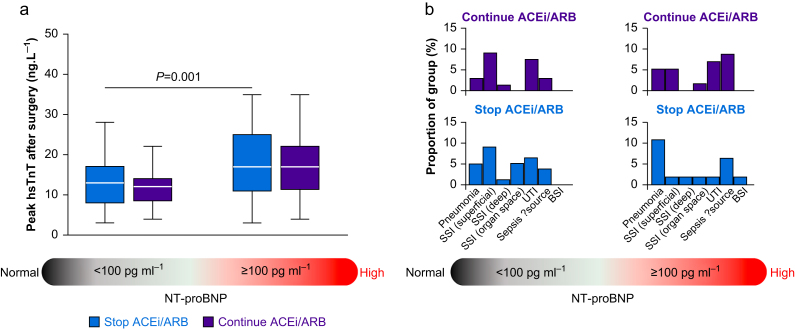
Secondary outcomes: peak high-sensitivity troponin and infectious complications. (a) Peak troponin values 24–48 h after surgery, stratified by NT-proBNP in subjects randomised to stop or continue renin–angiotensin system (RAS) inhibitors. (b) Sources of infection stratified by NT-proBNP in subjects randomised to stop or continue RAS inhibitors. For subjects with preoperative NT-proBNP <100 pg ml^−1^, 24/75 (32%) who stopped RAS inhibitors had an infectious complication within 30 days of surgery, compared with 16/65 (24.6%) who continued RAS inhibitors (odds ratio for stopping 1.44; 95% confidence interval 0.69–3.03). For preoperative NT-proBNP ≥100 pg ml ^−1^, 13/45 (28.9%) subjects who stopped RAS inhibitors had a postoperative infection, compared with 16/56 (28.6%) who continued RAS inhibitors (odds ratio for stopping 1.02; 95% CI 0.43–2.42). ACEi, angiotensin converting enzyme inhibitor; ARB, angiotensin-II receptor blocker; BSI, bloodstream infection; hsTNT, high-sensitivity troponin-T; NT-proBNP, N-terminal pro-B-type natriuretic peptide; SSI, surgical site infection; UTI, urinary tract infection.

For subjects with preoperative NT-proBNP <100 pg ml^−1^, the mean difference in peak troponin between the stop and continue groups was 1 ng L^−1^ (95% CI −2 to 4 ng L^−1^). For subjects with preoperative NT-proBNP ≥100 pg ml^−1^, mean difference in peak troponin between stop and continue groups was 4 ng L^−1^ (95% CI −5 to 13 ng L^−1^; Fig. 3a). There was no correlation between preoperative NT-proBNP concentrations and peak troponin within 48 h after surgery (Supplementary Fig. S4).

#### Infection

While 49/241 (20.3%) subjects sustained infectious complications after surgery (Fig. 3b), the incidence of all-cause infections was similar between the stop and continue groups independent of preoperative NT-proBNP concentrations (*P*=0.89). For subjects with preoperative NT-proBNP <100 pg ml ^−1^, 24/75 (32.0%) who stopped RAS inhibitors had an infectious complication within 30 days of surgery, compared with 16/65 (24.6%) who continued RAS inhibitors (OR for stopping 1.44; 95% CI 0.69–3.03). For preoperative NT-proBNP ≥100 pg ml ^−1^, 13/45 (28.9%) patients who stopped RAS inhibitors had postoperative infection, compared with 16/56 (28.6%) who continued RAS inhibitors (OR for stopping 1.02; 95% CI 0.43–2.42).

#### Major adverse cardiovascular events

Clinically diagnosed myocardial infarction, stroke, or death were recorded in fewer than 10 participants.

#### STEP-COMPAC core outcome set

**Fig 4:**
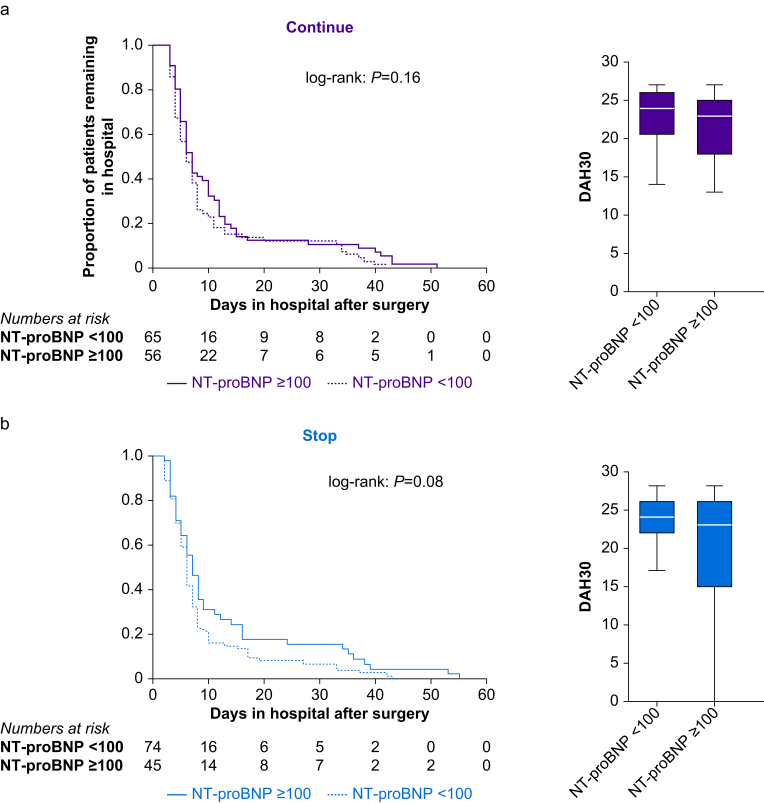
Length of hospital stay. (a) Kaplan–Meier plot showing duration of hospital stay and DAH30 for subjects randomised to continue renin–angiotensin system (RAS) inhibitors, stratified by NT-proBNP ≥100 pg ml^−1^ or NT-proBNP <100 pg ml ^−1^. (b) Kaplan–Meier plot showing duration of hospital stay and DAH30 for patients randomised to stop RAS inhibitors, stratified by NT-proBNP ≥100 pg ml^−1^*vs* NT-proBNP <100 pg ml^−1^. *P*-values refer to log-rank test. NT-proBNP, N-terminal pro-B-type natriuretic peptide.

Duration of hospital stay and DAH30 were similar for subjects who continued (Fig. 4a) or stopped RAS inhibitors (Fig. 4b). There were 21/241 (8.3%) subjects readmitted to hospital after surgery. EQ5D™ was also not related to preoperative NT-proBNP or management of RAS inhibitors (Supplementary Table S2).

## Discussion

The principal finding of this predefined substudy of the SPACE phase 2a RCT is that stopping RAS inhibitors in patients at lower perioperative risk (preoperative NT-proBNP <100 pg ml^−1^) was associated with a two-fold risk of sustaining MINS. For individuals with higher risk of complications after surgery (preoperative NT-proBNP ≥100 pg ml^−1^), the incidence of myocardial injury was similar between patients who stopped or continued RAS inhibitors during the perioperative period. These data indicate that lower-risk patients might benefit from continuing RAS inhibitors during the perioperative period.

We confirmed that NT-proBNP is a robust indicator for postoperative complications, as reported by both the VISION consortium^11^ and METS.^13^ Compared with preoperative NT-proBNP <100 pg ml^−1^, as adopted by our study, progressively higher NT-proBNP threshold values were associated with a dose–response increase in the incidence of vascular death or MINS within 30 days after surgery.^11^ NT-proBNP values enhanced the clinical stratification calculated from the Revised Cardiac Risk Index, with >25% subjects reassigned to a different risk strata.^11^ Although a limitation of the VISION study was the need for further external validation, our data suggest that using the very same NT-proBNP thresholds identified by VISION are robust and generalisable.

Given that our substudy shows that preoperative arterial pressure was similar between lower-risk patients, our data suggest that abrupt withdrawal of ACEi and ARBs can increase myocardial injury through mechanisms other than blood pressure control. Cessation of ACEi and ARBs could promote cellular injury through numerous mechanisms driven by the pathophysiological actions of angiotensin-II. Firstly, nicotinamide adenine dinucleotide phosphate (NADP) oxidase directly generates reactive oxygen species (ROS) after activation by circulating angiotensin-II.^30^ A vicious cycle of ROS-induced ROS release from damaged mitochondria and uncoupled nitric oxide synthase promotes cellular injury across disparate organs. Secondly, angiotensin-II upregulates the ubiquitous pro-inflammatory TLR4 receptor.^31^ Moreover, our data are consistent with a study that found that withdrawal of ACEi and ARB after hospitalisation for acute heart failure with reduced ejection fraction was associated with higher rates of post-discharge mortality or readmission, independent of the severity of heart failure.^15^ It might be that higher-risk patients, as indicated by preoperative NT-proBNP ≥100 pg ml^−1^, have limited cellular reserves to mitigate the adverse consequences of acute increases in circulating angiotensin-II. This hypothesis is consistent with the mosaic theory, whereby numerous pathophysiological mechanisms converge to prevent the integrative host response to injury and inflammation limiting organ injury.

Our study reinforces the need for objective biomarkers in understanding perioperative cardiovascular risk, not least because current European^12^^,^^18^ and North American^19^^,^^20^ guidelines do not provide specific advice on this issue. The METS study demonstrated that a substantial number of patients have unsuspected high NT-proBNP concentrations before surgery, which are consistent with occult heart failure.^13^^,^^21^ In-depth cardiovascular phenotyping of patients with colorectal cancer before surgery identified similarly impaired cardiovascular function, independent of whether patients received preoperative chemotherapy.^32^ The latest ESC (2022) guidelines state that ‘Data on perioperative use of renin–angiotensin–aldosterone system (RAAS) inhibitors are inconclusive’.^18^ The American Heart Association/American College of Cardiology guidelines (2021) concurred stating that ‘Temporary preoperative withholding of RAS inhibitors may also reduce MINS, but the available evidence is limited to data from observational studies.’^19^ The management guidelines from European Society of Anaesthesiology and Intensive Care (ESAIC: 2014) suggested that ‘When LV dysfunction is discovered during preoperative evaluation in untreated patients in a stable condition, surgery should be postponed, if possible, to allow for diagnosis of the underlying cause and the introduction of ACEIs and beta-blockers’.^33^ The ESAIC preoperative assessment guideline on biomarkers did not specifically address heart failure or ACE and ARBs.^12^ The Canadian Cardiovascular Society guidelines on perioperative cardiac risk assessment and management for patients who undergo noncardiac surgery recommends to ‘Withhold ACEI/ARB 24 hours before noncardiac surgery and restart ACEI/ARB on day 2 after surgery, if the patient is hemodynamically stable.’^20^ The latter recommendation did not account for pharmacokinetic characteristics of ACEi and ARBs.

Our data suggest that ongoing pragmatic multicentre trials examining the management of RAS inhibitors in patients age ≥18 yr undergoing noncardiac surgery should consider using natriuretic peptide biomarkers to refine interpretation of their data.^34^, ^35^, ^36^ Because two large database studies in noncardiac^37^ and cardiac surgery^38^ have identified that failure to restart RAS inhibitors is strongly associated with excess morbidity and mortality, further work is required to establish whether serial NT-proBNP measurements can help guide recommencing this drug class. This is particularly relevant given that acute hypertension is independently associated with myocardial injury,^39^ most likely through elevated preload causing stretch-induced cardiomyocyte stress.^40^

Strengths of our study are that the NTproBNP assays were batch analysed under the same conditions by investigators masked to both treatment allocation and troponin values. We used troponin thresholds to define myocardial injury that are broadly recommended by the VISION study,^1^ American Heart Association/American College of Cardiology,^19^ recent ESC,^18^ and STEP-COMPAC^24^ guidelines. However, we did not establish the aetiology of increased troponin-T concentrations by electrocardiographic criteria; whereas this might be instructive, the appearance of elevated troponin early in the postoperative period is associated with poorer outcomes regardless of aetiology.^4^ The lack of serial BNP measurements was a limitation, as postoperative BNP increase is also independently associated with adverse cardiac events after noncardiac surgery.^41^ Although patients with a clinical diagnosis of cardiac failure comprised <10% of our trial population, more granular echocardiographic data might have provided more insights as to why ∼40% of SPACE patients had NT-proBNP concentrations approaching or exceeding those indicative of structural heart disease or cardiac failure.^42^ Over a 10-yr period of serial BNP sampling, increasing age, new myocardial infarction and increased left atrial diameter, left ventricular end-diastolic diameter, and left ventricular mass index were associated with higher BNP concentrations. Thus, although the interpretation of NT-proBNP requires several covariates to be considered, significant pathophysiologic alterations that are likely to affect perioperative cardiovascular function adversely are evident at these higher levels.

In conclusion, discontinuation of renin–angiotensin system inhibitors in lower-risk patients undergoing noncardiac surgery increased the risk of myocardial injury or other complications.

## Authors’ contributions

ELISA measurements and analysis: AGDA

Statistical analysis: AP

Trial management: TEFA, SB, PD

Subject enrolment and data collection: SS, AM, SC, DB

RCT design: RMP

Conception, RCT and substudy design, analysis: GLA

## Declaration of interest

RMP, TEFA, GLA are editorial board members of the *British Journal of Anaesthesia*. The other authors declare that they have no conflicts of interest.

## Funding

British Oxygen Company research chair grant from the 10.13039/501100001297Royal College of Anaesthetists, administered by the 10.13039/501100000349National Institute for Academic Anaesthesia (GLA) and UK National Institute for Healthcare Research CLRN Portfolio. GLA supported by 10.13039/501100000272NIHR Advanced Fellowship (NIHR300097) and 10.13039/501100000274British Heart Foundation programme grant (RG/19/5/34463).
