# Supplementary material for: Preoperative N-terminal pro-B-type natriuretic peptide and myocardial injury after stopping or continuing renin–angiotensin system inhibitors in noncardiac surgery: a prespecified analysis of a phase 2 randomised controlled multicentre trial
Source: Br J Anaesth. 2024 Feb 9;132(5):857–66. doi: 10.1016/j.bja.2024.01.010 (PMC11103084; doi:10.1016/j.bja.2024.01.010)
Supplement: Multimedia component 1 [file mmc1.docx]

**Preoperative NT-proBNP and myocardial injury after stopping or continuing renin–angiotensin system inhibition in noncardiac surgery: pre-specified analysis of a phase-II, randomised controlled multi-centre trial.**

Supplementary data.

Contents

[Study Investigators list 2](#_Toc154227055)

[Independent steering committee 2](#_Toc154227056)

[Schedule of events 3](#_Toc154227057)

[Supplementary figure 1: NT-proBNP standard curves. 4](#_Toc154227058)

[Supplementary figure 2: National Institute for Health and Care Excellence (NICE) defined bands indicating NT-proBNP threshold values for heart failure – relationship with primary outcome myocardial injury within 48h of surgery. 5](#_Toc154227059)

[Supplementary Figure 3. NT-proBNP and myocardial injury in patients with hsTnT >15ng/L before surgery. 6](#_Toc154227060)

[Supplementary figure 4: NT-proBNP – correlation with peak hsTnT within 48h of surgery. 7](#_Toc154227061)

[Supplementary Table 1: All cause complications. 8](#_Toc154227062)

[Supplementary Table 2. NT-proBNP, myocardial injury and EQ5D™ 30 days after surgery. 9](#_Toc154227063)

# Study Investigators list

#

*The Royal London Hospital:* Gareth Ackland (Local coordinator), Tim Martin, Maria Fernandez, Fatima Seidu, Mari-Liis Pakats, Otto Mahr, Neil MacDonald, Filipa Dos Santos, Amaia Arrieta Garcia, Ruzena Uddin, Salma Begum, Rupert Pearse, Emily Subhedar, Yize Wan, Akshaykumar Patel, Tasnin Shahid, Mevan Gooneratne, Charlotte Trainer, Bethan Griffiths, Steven Dunkley, Shaun May, Sophie Walker, Alexander Fowler, Timothy Stephens, Monica Oliveira, Marta Januszewska, Edyta Niebrzegowska, Vanessa Amaral, Jamila Kassam, Sophie Young, Shanaz Ahmad, Jan Whalley, Ryan Haines, Sara Hui, Rob Hammond, David Crane.

*University College Hospitals:* David Brealey (Local coordinator), Sohail Bampoe, Robert Stephens, Anna Reyes, Gladys Martir, Chimverly Diaz

*Derriford Hospital Plymouth:* Stuart Cleland (co-Local coordinator), Gary Minto (co-Local coordinator), Natasha Wilmshurst, Debbie-Claire Affleck, Tracy Ward, Gavin Werrett, Susan Cummins, Alan Amber, Andrew Biffen, Stephen Boumphrey, Elizabeth Cann, Charlotte Eglinton, Elaine Jones, Memory Mwadeyi, Sam Piesley, Richard Cowan, Julie Alderton, Fiona Reed, Joanne Smith, Amy Turner, Lorraine Madziva, Abigail Patrick, Penny Harris, Harry Lang

*Bristol Royal Infirmary:* Alexander Middleditch (Local coordinator), Anthony Pickering, Catherine O’Donovan, Rebecca Houlihan, Rosina Jarvis, Andrew Shrimpton, Toni Farmery, Katy Tucker, Danielle Davis

*University Hospital of North Durham:* Sameer Somanth (Local coordinator), Louise Duncan, Helen Melsom, Sarah Clark, Melanie Kent, Michelle Wood, Ami Laidlaw, Tracy Matheson-Smith, Kathryn Potts, Andrea Kay, Stefanie Hobson.

# Independent steering committee

Chair: John Sear (Oxford, UK). Members: Vikas Kapil (clinical pharmacologist/internist, QMUL), Andrew Archbold (cardiology, Barts Heart Centre, Barts Health NHS Trust) Matt Wilson (perioperative trials, Univ Sheffield); Drilona Dndrejaj (lay member); Dennis Ly (lay member) Akshaykumar Patel (study statistician)

# Schedule of events

| **Event/Visit** | **Screening** | **Pre-op 72 hrs before surgery** | **Pre-op ≥ 48 hrs before surgery** | **Day of surgery** | **Postop day 1 ± 6 hrs** | **Postop day 2 ± 6 hrs** | **Postop day**  **3 ± 6 hrs** | **30 days after surgery** |
| --- | --- | --- | --- | --- | --- | --- | --- | --- |
| Inclusion/exclusion criteria | x |  |  |  |  |  |  |  |
| Informed consent | x |  |  |  |  |  |  |  |
| Demographic information | x |  |  |  |  |  |  |  |
| Medical history | x |  |  |  |  |  |  |  |
| Prior and concomitant medications | x |  |  | x | x | x | x | x |
| Height and weight |  |  |  | x |  |  |  |  |
| EQ-5D-3L | x |  |  |  |  |  |  | x |
| Planned level of care | x |  |  |  |  |  |  |  |
| Level of care |  |  |  | x | x | x | x |  |
| Randomisation |  | x |  |  |  |  |  |  |
| Perioperative information |  |  |  | x |  |  |  |  |
| Review of medical notes | x |  | x |  | x | x | x | x |
| Restart drug |  |  |  |  |  | x |  |  |
| Blood sample |  |  | x* | x | x | x |  |  |
| Hemoglobin † | x† |  |  |  |  |  |  |  |
| Creatinine † | x† |  |  | x | x | x | x |  |
| Blood pressure/ heart  rate^s^ |  |  |  | x | x | x |  |  |
| Intravenous fluids |  |  |  | x | x | x | x | x |
| Telephone contact |  |  | x |  |  |  |  | x |
| Review of AE/SAE |  |  |  | x | x | x | x |  |
| End of trial form |  |  |  |  |  |  |  | x |

# Supplementary figure 1: NT-proBNP standard curves.

Summary of standard values for each plate used for batched ELISA analysis of plasma NT-proBNP.


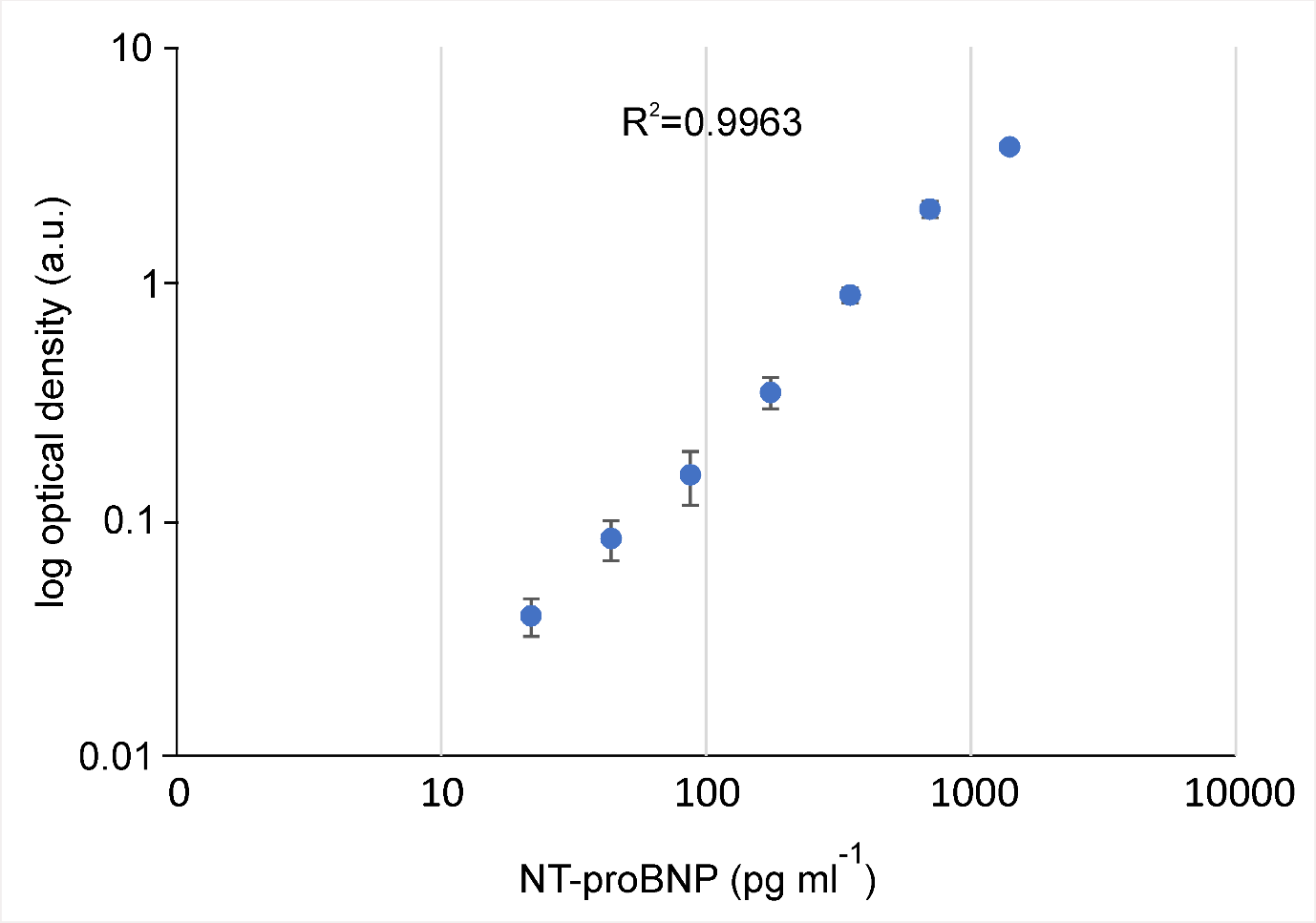


# Supplementary figure 2: National Institute for Health and Care Excellence (NICE) defined bands indicating NT-proBNP threshold values for heart failure – relationship with primary outcome myocardial injury within 48h of surgery.

Numbers in bars indicate total number of patients in each category.


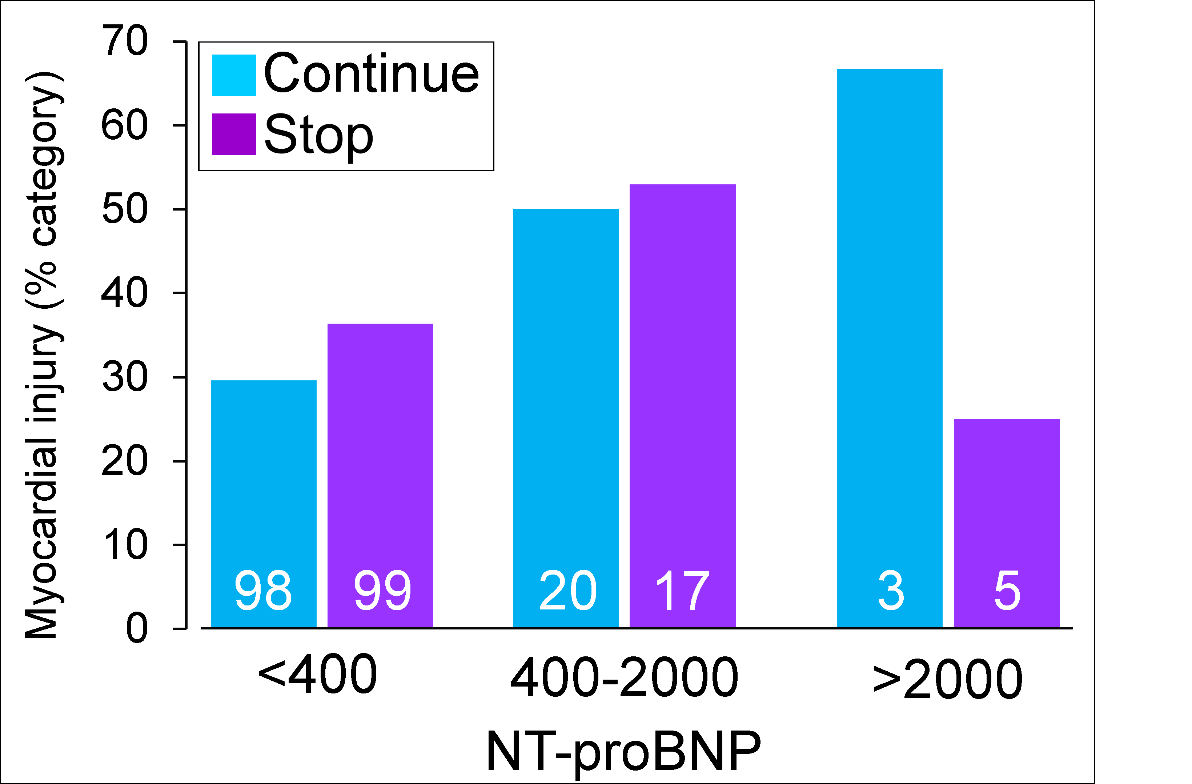


# Supplementary Figure 3. NT-proBNP and myocardial injury in patients with hsTnT >15ng/L before surgery.

Numbers in bars refer to total in each category.


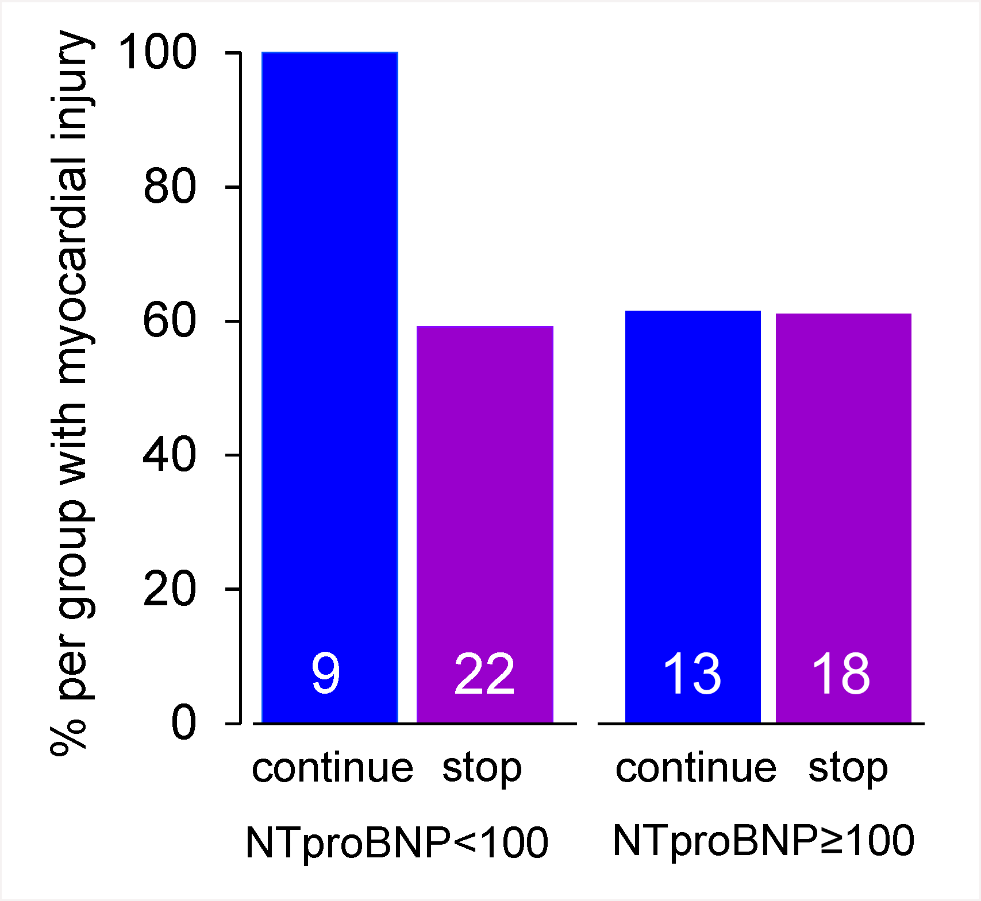


# Supplementary figure 4: NT-proBNP – correlation with peak hsTnT within 48h of surgery.

Inset graph shows maximal hsTnT for entire range of NT-proBNP (maximal value 3100pg.ml^-1^).


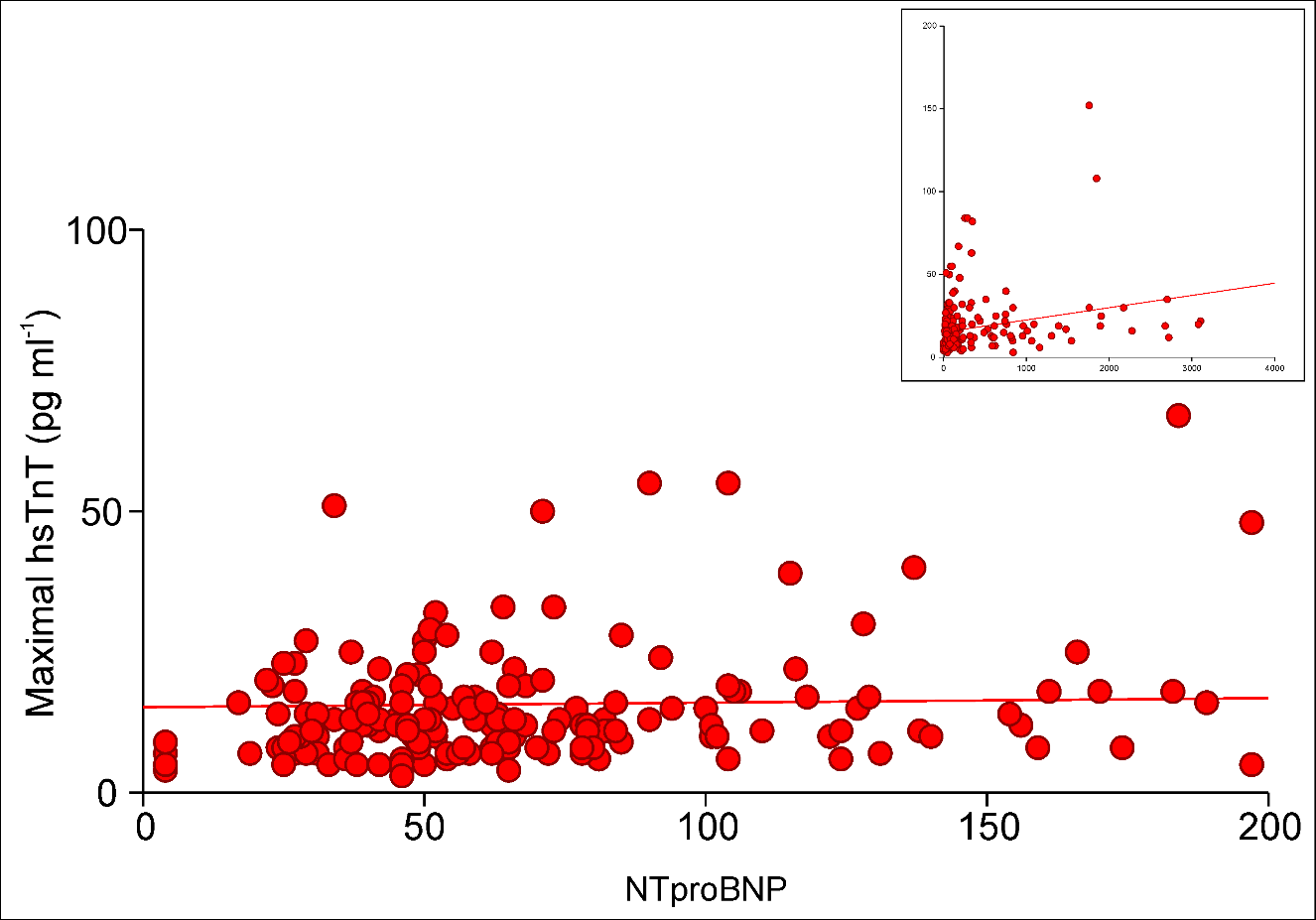


# Supplementary Table 1: All cause complications.

NT-proBNP units: pg ml^-1^.

|  | **Stop** | | **Continue** | |
| --- | --- | --- | --- | --- |
|  | **NT-proBNP < 100** | **NT-proBNP ≥100** | **NT-proBNP < 100** | **NT-proBNP ≥100** |
|  | **n=75** | **n=45** | **n=65** | **n=56** |
| **Complication** |  |  |  |  |
| Cardiac - no. (%) |  |  |  |  |
| Arrhythmia | 2 (2.7) | 2 (4.4) | 0 (0.0) | 3 (5.4) |
| Cardiac arrest with successful resuscitation | 0 (0.0) | 1 (2.2) | 0 (0.0) | 0 (0.0) |
|  |  |  |  |  |
| Respiratory - no. (%) |  |  |  |  |
| Pneumonia | 4 (5.3) | 5 (11.1) | 2 (3.1) | 3 (5.4) |
| Pleural effusion | 3 (4.0) | 0 (0.0) | 0 (0.0) | 1 (1.8) |
| Pneumothorax | 0 (0.0) | 0 (0.0) | 0 (0.0) | 1 (1.8) |
| Bronchospasm | 1 (1.3) | 0 (0.0) | 0 (0.0) | 0 (0.0) |
| Aspiration pneumonitis | 0 (0.0) | 0 (0.0) | 0 (0.0) | 0 (0.0) |
| Acute lung injury | 0 (0.0) | 0 (0.0) | 0 (0.0) | 0 (0.0) |
| Acute respiratory distress syndrome | 0 (0.0) | 0 (0.0) | 0 (0.0) | 0 (0.0) |
| Infection - no. (%) |  |  |  |  |
| Surgical site infection (superficial) | 7 (9.3) | 1 (2.2) | 6 (9.2) | 3 (5.4) |
| Surgical site infection (deep) | 1 (1.3) | 1 (2.2) | 1 (1.5) | 0 (0.0) |
| Surgical site infection (organ space) | 4 (5.3) | 1 (2.2) | 0 (0.0) | 1 (1.8) |
| Urinary tract infection | 5 (6.7) | 1 (2.2) | 5 (7.7) | 4 (7.1) |
| Infection, source uncertain | 3 (4.0) | 3 (6.7) | 2 (3.1) | 5 (8.9) |
| Laboratory confirmed bloodstream infection | 0 (0.0) | 1 (2.2) | 0 (0.0) | 0 (0.0) |
|  |  |  |  |  |
| Other - no. (%) |  |  |  |  |
| Pulmonary embolism | 0 (0.0) | 0 (0.0) | 1 (1.5) | 0 (0.0) |
| Acute psychosis or delirium | 1 (1.3) | 1 (2.2) | 1 (1.5) | 2 (3.6) |
| Bowel infarction | 1 (1.3) | 0 (0.0) | 0 (0.0) | 0 (0.0) |
| Anastomotic leak | 1 (1.3) | 0 (0.0) | 0 (0.0) | 3 (5.4) |
| Perforation of viscus | 0 (0.0) | 0 (0.0) | 0 (0.0) | 0 (0.0) |
| Gastro-intestinal bleed | 0 (0.0) | 2 (4.4) | 0 (0.0) | 0 (0.0) |
| Other postoperative haemorrhage | 0 (0.0) | 0 (0.0) | 1 (1.5) | 0 (0.0) |
| Any other complication | 11 (14.7) | 14 (31.1) | 14 (21.5) | 10 (17.9) |
| Acute kidney injury | 10 (13.3) | 8 (17.8) | 11 (16.9) | 11 (19.6) |

# Supplementary Table 2. NT-proBNP, myocardial injury and EQ5D™ 30 days after surgery.

| <100pg.ml-1 | | | >100pg.ml-1 | |
| --- | --- | --- | --- | --- |
|  | No myocardial injury | Myocardial injury | No myocardial injury | Myocardial injury |
| Mobility |  |  |  |  |
| *No problems* | 23 | 19 | 26 | 17 |
| *Some problems* | 39 | 29 | 43 | 24 |
| *Confined to bed* | 2 | 1 | 2 | 0 |
| Self_care | | |  |  |
| *No problems* | 45 | 36 | 46 | 29 |
| *Some problems* | 15 | 10 | 19 | 12 |
| *Unable to wash/dress* | 4 | 3 | 6 | 0 |
| Daily activities | | |  |  |
| *No problems* | 24 | 22 | 27 | 16 |
| *Some problems* | 29 | 22 | 32 | 19 |
| *Unable to perform daily activities* | 11 | 5 | 12 | 6 |
| Pain | |  |  |  |
| *No pain/discomfort* | 24 | 17 | 20 | 14 |
| *Moderate pain/discomfort* | 33 | 29 | 45 | 25 |
| *Extreme pain/discomfort* | 7 | 3 | 6 | 2 |
| Anxiety/depression | |  |  |  |
| *Not anxious/depressed* | 43 | 38 | 53 | 33 |
| *Moderately anxious/depressed* | 18 | 8 | 16 | 8 |
| *Extremely anxious/depressed* | 3 | 3 | 2 | 0 |
| Health score |  |  |  |  |
| mean | 72 | 70 | 69 | 72 |
| SD | 17 | 19 | 18 | 21 |
